# Individualized support for informal caregivers of people with dementia – effectiveness of the German adaptation of REACH II

**DOI:** 10.1186/s12877-017-0678-y

**Published:** 2017-12-12

**Authors:** Martin Berwig, Stephanie Heinrich, Jenny Spahlholz, Nina Hallensleben, Elmar Brähler, Hermann-Josef Gertz

**Affiliations:** 1 Clinic and Policlinic for Psychiatry and Psychotherapy, University of Leipzig, Leipzig, Germany; 2 Medical Faculty, Leipzig, Germany; 3 German Center for Neurodegenerative Diseases (DZNE) – Site Witten, Witten, Germany; 4 Faculty of Health, University Witten/Herdecke, Witten, Germany; 5 Department of Rehabilitation Medicine, Medical Faculty, Martin Luther University of Halle-Wittenberg, Halle, Germany; 6 Department of Medical Psychology and Medical Sociology, Medical Faculty, University of Leipzig, Leipzig, Germany; 7 Clinic and Policlinic for Psychosomatic Medicine and Psychotherapy, Medical Faculty, University of Mainz, Mainz, Germany

**Keywords:** Dementia, Informal caregiver, Burden, Multicomponent intervention, REACH II

## Abstract

**Background:**

Individualized, outreach and structured multicomponent interventions are a promising intervention approach to relieve the burden of informal caregivers of people with dementia. In this study, we adapted and evaluated a multicomponent intervention (Resources for Enhancing Alzheimer’s Caregiver Health II, REACH II), which was developed in the USA, to the German health-care system. Therefore the project is called the German adaptation of REACH II (in German: Deutsche Adaptation der REACH II, DE-REACH).

**Methods:**

The effectiveness of DE-REACH was examined in a randomized, controlled trial on 92 informal caregivers of people with dementia. The intervention comprised 12 individual two-weekly sessions (9 at home with the informal caregiver and 3 via telephone) and combined five modules. The reduction of the burden of the informal caregivers was chosen as the primary outcome.

**Results:**

The results showed a great stabilizing effect of the intervention on caregiver burden (effect size d = 0.91), that is, comparing pre- and post-measurements the burden decreased very slightly in the intervention group whereas it increased very strongly in the control group. After a three-month follow-up period this effect decreased from a great to a moderate effect. There were also improvements as a result of the intervention in somatization, health-related psychological quality of life and the reaction of the informal caregivers in response to challenging behaviors of the relative with dementia. Moreover, the frequency of challenging behaviors of the affected person itself was reduced in favor of the intervention.

**Conclusion:**

The findings of this study provide further evidence for the impact of multicomponent support interventions for informal caregivers of people with dementia.

**Clinical trial registration:**

NCT01690117. Registered September 17, 2012.

## Background

Informal caregivers of people with dementia often show a higher stress level than that of caregivers of physically frail elderly people [1], whereby the conditions of the burden experience are complex [2]. Obviously non-cognitive symptoms of dementia, for instance psychotic symptoms, depression and challenging behaviors, are of particular importance [3, 4]. Caring for a person with dementia is time-consuming and associated with significant personal engagement and day-to-day management. Due to these high demands and high level of burden there is a higher risk of falling physically and mentally ill for informal caregivers of people with dementia [5].

Hitherto, a wide range of psychosocial interventions have been developed, which were targeted to enhance the mental and physical health of the informal caregivers and to reduce their experience of burden (e.g. [1]). These interventions differ in terms of their format (individual or group format) and content (psycho-education; assessment of symptoms; problem solution; training of abilities; strategies for coping with burden; behavior modification). In addition, all these formats are offered not only as face-to-face interventions but also as proxy interventions by using the telephone [6] or Internet [7]. Systematic reviews and meta-analyses [5, 8, 9] prove that the impact of an intervention is affected by the selected format and content of the intervention. Individualized programs are proved to be more effective than group interventions, problem-solving strategies and behavior modification are shown to be more effective than psycho-education and solely knowledge transfer. Moreover, structured multicomponent interventions are possibly able to delay or prevent the institutional care of the person with dementia [5]. A meta-analysis on the REACH I (Resources for Enhancing Alzheimer’s Caregiver Health I) project concludes that in terms of burden and depression, individualized, outreach and structured multicomponent interventions are the most effective [10]. This conclusion is confirmed by the results of two actual meta-reviews or analyses, respectively [11, 12].

### Theoretical background of REACH II

**Fig. 1:**
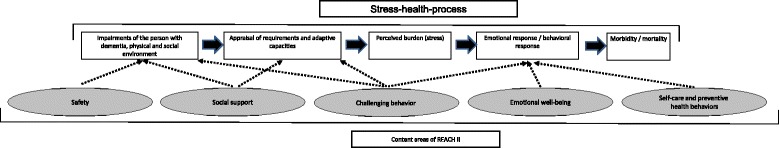
Supposed impact of the different intervention components on the stress–health process of the informal caregiver (adapted from Schulz [14])

REACH I was the first step of a comprehensive program for the development of effective measures to support informal caregivers of people with dementia in the USA, where several intervention components were tested, evaluated and compared with each other [10, 13]. On the basis of the results of REACH I, an outreach, intensive and individualized multicomponent intervention was designed as a second step of this program, hence it is called REACH II. The REACH II intervention is based on the theoretical framework model for the stress–health process of informal caregivers of people with dementia according to Schulz [14]. With regard to this model, perceived burden (stress), the corresponding (emotional) behavioral response and finally morbidity and mortality of the informal caregiver, respectively, depend on the appraisal of the objective care situation (e.g. impairments of the person with dementia, physical and social environment) and the respective individual adaptive resources. These are, for example, the ability to care for a person with dementia and to cope with the demands of the care situation. Individually pronounced factors of the informal caregiver moderate the three main components of the stress–health process (objective care situation, appraisal of adaptive resources, emotional reaction) and could therefore provide a starting point for an intervention (see Fig. 1) [14]. These areas are: 1. Safety; 2. social support; 3. challenging behaviors; 4. emotional well-being; 5. self-care and preventive health behaviors.

For example, if the living environment has become safer for the person with dementia, the caregiver needs to worry lesser, it may be even possible to leave the affected person alone for a short time. In other words, the requirements of the care situation for the caring relative have become lower in this regard. Another possibility to lower demands of the care situation may result from reducing the frequency of challenging behaviors by modifying the upholding conditions of the behaviors. When I feel socially supported, because, for example, the person with dementia visits daytime care for people with dementia once or more often a week, this will positively influence the appraisal of my adaptive resources. There will be the same adaptation-enhancing effect for the informal caregiver by coping in a more relaxed way with challenging behaviors of the person with dementia. The negative psychophysical effects of the emotional stress reaction can be diminished, for example, if the informal caregiver succeeds in pursuing some pleasant activities every week, or even by using relaxing techniques.

### Description of the original REACH II program

The REACH II program is an individualized, psycho-educational, and skills-training evidence-based multicomponent intervention, which aims to reduce risk in five caregiver domains (see above) by developing caregivers’ ability to cope with the challenging behaviors of the relative with dementia and providing social support, cognitive behavioral restructuring strategies to modify negative emotional responses, and strategies for enhancing healthy behaviors [15]. It is delivered by certified interventionists with at least a bachelor’s degree, takes place over six months, and includes 12 sessions (9 in-home [1.5 h each] sessions and 3 telephone [half-hour each] sessions) (see Table 1) and five structured telephone support group sessions (once a month).

**Table 1:** Structure of the DE-REACH intervention

Session	Week	Type of Contact
1	1	home visit
2	2	home visit
3	3	home visit
4	4–5	home visit
5	6–7	home visit
6	8–9	home visit
7	11	telephone call
8	13–14	home visit
9	16	telephone call
10	17–18	home visit
11	20	telephone call
12	21–22	home visit

The intervention involves a range of strategies [15]: information transfer, psycho-education, role-playing, problem-solving, practice of skills, techniques for stress management, and telephone support groups. Notebooks with educational materials are handed out to the participants to support the intervention process and each session builds on one another and includes a reflection of the themes discussed in the previous session (for an exemplary structure of an intervention session see Table 1 of the protocol for this study [16]). Belle and colleagues [15] argue that each care situation differs inherently, and this is the reason why it should be possible to adapt an intervention to the specific needs of the individual. Therefore, at the beginning of the REACH II intervention an individual risk assessment of five major caregiver domains (knowledge of dementia, ability to care, perceived social support, emotional and physical well-being and challenging behavior) is conducted. Based on this assessment, individual treatment strategies were selected (see Table 2).

**Table 2:** Addressed domains of the DE-REACH intervention and exemplary assignment of the risk appraisal and treatment strategies (from Heinrich [16])

Domain	Session	Examples of Risk Appraisal	Examples of Treatment Strategies
Security	2	- dangerous objects are at home of person with dementia - wandering of the person with dementia - possibility of leaving the relative with dementia alone at home	- removal and locking away of dangerous objects - using opportunities for identification - designing the living space - determining measurements (e.g. installing smoke-detectors) - eliminate root causes for wandering
Social support	1–2	- support and help of friends - exchange in carer support groups - possibility of care break	- explanation of the importance of social support - referral to support groups located nearby - application of rehabilitation measures - counselling to respite services
Challenging behaviors	3–10	- lack of knowledge of symptoms of dementia - experience of burden in coping with activities of daily life or physical care of the relative with dementia, respectively	- providing information about dementias - observation and analysis of behaviours and working out a problem-solving process - training of various techniques, role playing or demonstrations
Emotional well-being	2–10	- experience of irritability, anger, frustration and stress states	- teaching in mood management - learning and training of relaxation techniques - cognitive restructuring
Self-care and preventive Health Behaviors	2–3	- sleeping problems - appraisal of physical health - meeting medical appointments and preventive measures	- teaching to cope with physical complaints - instructions on physical activity - medical appointments based on the ’medical book’ - self-monitoring and overview of existing diseases, drugs and examinations

The initial two in-home sessions serve to explain the intervention process to the participants and to begin tailoring the intervention on the basis of the risk assessment. Moreover, basic information on the importance of self-care and techniques to improve healthy behaviors are communicated. Furthermore, caregivers receive a ‘Medical Book’ [17], which is intended to facilitate self-organization and scheduling of health maintenance activities of the caregivers (such as medical check-ups). A central aim of the remaining intervention visits is to develop the caregiver’s ability to cope with challenging behaviors of the relative with dementia and their own reaction in response to these behaviors. This should lead to an improved well-being [15]. Hence, in line with techniques of behavior therapy, a problem-solving process, covering several sessions, is initiated (including defining problems in specific and objective terms, translating problems into objective goals, and generating specific action-oriented steps to solve the problems), whereby a maximum of three identified problems are worked on during the intervention process. Moreover, also in line with techniques of behavior therapy, skills training, techniques for stress mood management, including breathing exercises, listening to music, stretching exercises and strategies for increasing involvement in pleasant events are integrated into the REACH II sessions. A detailed description of the original REACH II intervention is available at the Epidemiology Data Centre of the University of Pittsburgh [18].

### Aims of the present study

REACH II was evaluated in a multicenter randomized controlled trial and showed an enhancement of health-related quality of life and a reduced prevalence of clinical depression in informal caregivers [15]. Despite its benefits and proven effectiveness it has not yet been applied in German-speaking areas.

First, this study aimed to translate and adapt the original REACH II program to the requirements of the German health-care system. Second, it aimed to evaluate the effectiveness of this German adaptation of REACH II (in German: Deutsche Adaptation der REACH II, DE-REACH), focusing on the burden of the informal caregivers. Burden was chosen as the primary outcome because in the theoretical framework of REACH II, according to Schulz [14] (see Fig. 1), this dimension is the central outcome and could be influenced by the intervention components.

According to the framework of the Medical Research Council (MRC) [19], this study is a phase III trial (evaluation).

## Methods

### Design

We used a randomized, controlled design (see Fig. 1 of the protocol for this study [16]). The intervention group received the DE-REACH intervention and the control group received usual care, whereby services of usual care corresponded to the available care services determined by the actual version of the German Care Insurance Law (e.g. caregiver counseling, utilization of day care, low-threshold care services, short-term or prevention care).

### Participants and recruitment

Eligibility criteria for informal caregivers included the following: age 21 years or older; living with or sharing cooking facilities with the care recipient; providing care for a relative with a medically diagnosed Alzheimer’s disease (AD) or related disorders, vascular dementia (VD) or behavior variant frontotemporal dementia (bvFTD) for at least 4 h per day for at least the past 6 months; informal caregiver speaks fluent German. We excluded caregivers who were involved in another caregiver intervention study, who had an actual psychiatric diagnosis of mental illness or another illness that would prevent 6 months of study participation, or the forthcoming institutionalization of the person being cared for. Other requirements were logistic, including having a telephone, and planning to remain in the geographic area for at least 6 months. Recruitment of participants was conducted in the first place by inviting informal caregivers, who sought consultations with their relatives with dementia for diagnostic and therapeutic purposes at the specialized outpatient clinic for dementia (memory clinic) of the University Hospital Leipzig, Department for Psychiatry and Psychotherapy, to participate. However, other measurements, such as project advertisements in local newspapers, informing local outpatient care services, local bureaus of statutory health insurances and senior centers, flyer distribution, etc. also took place. The ethical committee of the University of Leipzig approved the study before enrollment of participants.

### Study procedures

Written informed consent was obtained from informal caregivers and their relatives with dementia before conducting study-related procedures. We used a central randomization via randomization lists realized by an online procedure of the Medical Faculty of the Ludwig Maximilians University of Munich [20]. To obtain the same number of subjects in the intervention and the control group, we used a randomized block design [21]. The outcome rater was not informed about the randomization code of a participant before opening a sealed envelope with the code inside after completion of the baseline assessment. The interviews of baseline investigation always took place at home with the caregivers and their relatives with dementia. All further interviews at post and follow-up were carried out by telephone. For this purpose, the informal caregivers had a room document, which had been handed out at baseline examination, with the response options for the respective questionnaires. Thus, the post and the follow-up interviews, apart from using the telephone as communication medium, did not differ from the baseline interview. All the members of the author team, except EB and HJG, collected data at all measurement points.

### Intervention

**Fig. 2:**
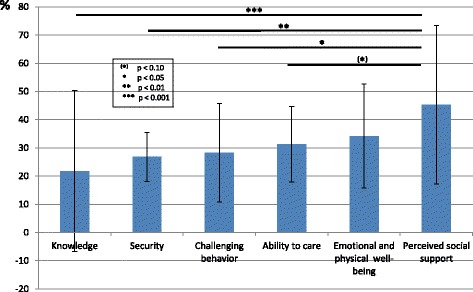
Mean percentage of items at risk for each risk domain (*N* = 41)

This German version of the original REACH II program (DE-REACH) had the same number of sessions and duration (6 months) and addressed the same care domains as REACH II. Likewise, the intervention has been tailored to the needs of each participant on the basis of his/her responses in the risk appraisal (see Fig. 2) and was delivered through active teaching techniques to the caregivers. The latter are techniques whereby the learner, or in our case the caregiver, is actively involved. This means, for example, that the caregiver actively transfers the learning contents of the counseling sessions to their everyday lives (e.g. relaxation technique strategies for coping with challenging behavior, etc.). We used most of the REACH II’s intervention and assessment protocols. A research assistant with outstanding English skills conducted the translations. The REACH II intervention is essentially based on the fundamentals and the repertoire of behavioral therapy. This approach to psychotherapy is very scientifically oriented and therefore to be seen as more or less culture-free. In other words, there is no specific German behavioral therapy. For this reason, we were of the opinion that an elaborate back-translation procedure could be dispensed with.

There were, however, three major differences between REACH II and this German version. First, accompanying structured telephone support group sessions and the specialized computer-integrated telephone system were not adopted because of technical and cost reasons. Results of three translational studies of REACH II showed that the intervention is effective even without either of these intervention components [22–24]. To compensate for the omission of the telephone-based support groups of the original REACH II intervention, we requested that each of the participants of the intervention group visit a local support group for informal caregivers of people with dementia. Another major difference consists in the fact that in Germany are other support measures that are financially supported by the social insurance system in Germany than those in the USA. In addition, the care advisory possibilities are organized differently. These were important details, which had to be adapted for the DE-REACH intervention. Therefore, in the consultation process of REACH II and in the caregiver notebook (see below) other help and offers of support were referenced.

Minor modifications were that the duration of the in-home sessions was reduced from 1.5 h to 1 h only (the duration of the half-hour telephone sessions was not changed) and that the required qualification for DE-REACH interventionists was a completed three-year health-care traineeship (e.g. occupational therapists, nurses) and professional experience in dementia care (requirements for interventionists of the original REACH II program see section “Description of the original REACH II program”).

Based on the caregiver notebook of REACH II, the research team produced an adapted version of the caregiver notebook for use by interventionists and informal caregivers during and beyond the intervention period. The caregiver notebook covers information about dementia, common caregiving difficulties, self-care, and community resources, compiled in a user-friendly format.

### Outcomes

#### Primary outcome

The primary outcome of the intervention was the change in the burden of the informal caregivers between the baseline and the post-intervention assessment. This was ascertained using an internationally widely used 22-item assessment tool for measuring the caregiver’s perceived burden (Zarit Burden Inventory ZBI), in which higher scores indicate higher burden [25, 26]. We applied a German version of the ZBI [26]. The primary outcome was assessed at baseline, after the completion of the intervention (month 6) and at follow-up 3 months later (month 9).

#### Secondary outcomes

Suitable primary outcomes should have a clinical relevance for the target group and be deducible from the respective theoretical intervention model. According to the Schulz model [14], the primary outcome for the present study is caregiver burden. The secondary target variables were selected in such a way that they additionally clinically characterize the primary outcome. In this way, they can, contribute to making a clinically significant primary outcome more trustworthy [27]. In accordance to this, all selected secondary outcomes for this study are related to the concept of burden (primary outcome) and were the changes between the baseline and the post-intervention assessments. These were the mental health of the informal caregiver, the ultra-short form of the Patient Health Questionnaire – 4 Items (PHQ-4) [28]; somatization of the informal caregiver, PHQ – module somatization (PHQ -15) [29]; health-related quality of life of the informal caregiver, short form of the SF- 36 Health Survey (SF-12) [30]; perceived social support of the informal caregiver, Enriched Social Support instrument (ESSI) [31]; frequency of challenging behaviors of the relative with dementia, frequency scale of the Revised Memory and Behavior Problem Checklist (RMBPC-24 frequency) [32]; reaction of the informal caregiver in response to challenging behaviors of the relative with dementia, reaction scale of the RMBPC-24 [32]. Apart from the RMBPC-24, we used validated German translations of the original scales for all secondary outcomes. Since there was no German adaptation available for the RMBPC-24, we prepared our own German version of that instrument. The forward and backward translation process was oriented to the guidelines for the cultural adaption of self-report instruments according to Beaton and colleagues [33]. Since there is no information available about the factorial structure of our German version of the RMBPC-24, only the total score was used. Potential long-term effects of the intervention were determined by analyzing changes in the primary outcome and the secondary outcomes from the baseline to the follow-up assessment. The primary outcome and the secondary outcomes were assessed at baseline and after the completion of the intervention (month 6). Secondary outcomes were also assessed at follow-up 3 months later (month 9). The patients’ cognitive ability (using the cognitive scale of the Structured Interview for Diagnosing Alzheimer-Type Dementia, Multi-Stroke Dementia, and Dementias of other Etiology according to DSM-III-R and ICD-10, SIDAM [34]) was recorded at baseline to confirm the presence of a clinical dementia syndrome in addition to the medical dementia diagnosis. Moreover, health-care service utilization of people with dementia was documented at baseline.

#### Sample size calculation

The calculation of the sample size was based on an updated meta-analysis determining the effectiveness of interventions for informal caregivers of older adults [8]. In that study, a moderate mean effect size of 0.65 (95% confidence interval (CI) [0.46–0.84,]) was determined for multicomponent interventions on caregiver burden. Assuming this effect size as significant for DE-REACH, which is a multicomponent intervention, and using a t-test for two independent samples with a 0.05 two-sided significance level, a sample size of 39 patients per group was calculated to have 80% power for detecting a treatment effect of that size (calculated by G*Power, [35]). Allowing for a dropout rate of 35%, a total of 53 informal caregivers per group was planned.

#### Quality management

Outcomes were assessed by independent raters who were kept blind to group assignment until baseline assessment was conducted. Independent raters were all at least graduated psychologists with a bachelor’s degree, and had much experience with all instruments used in the DE-REACH study. Interventionists were trained in intensive two-day training seminars. The quality and homogeneity of the study procedures, data collection, and treatment delivery were ensured by regular supervision of all study personnel. Standardization of the intervention was achieved by the adapted REACH II interventionist manual, which included a detailed description of each session. Process quality was documented by structured interventionist protocols of each problem-solving process (see above). Moreover, implemented content areas of the DE-REACH intervention program were recorded at follow-up.

#### Statistical analysis

The change from baseline to post-intervention assessment on the ZBI (primary outcome) was compared between the treatment groups using t-tests for independent samples on all randomized patients (intention-to-treat population). In the case of significant between-group differences at baseline, analysis of covariance was used with the post-intervention ZBI score as a dependent variable, and treatment group and baseline ZBI score as additional covariates. Several methods with missing value imputation were used, including expectation maximization method and the multiple imputations strategy implemented in the Statistical Packages for Social Sciences (SPSS) software version 20.0. Secondary analyses of the primary outcome variable were performed on patients treated per protocol (PP population). Secondary study outcomes were subjected to an explorative analysis. All *P* values were two-sided with a significance level of 0.05. Secondary analyses were explorative without multiplicity adjustment. Moreover, a statistical subgroup analysis was calculated only for those cases where the presence of dementia syndrome was confirmed by the SIDAM score (SISCO) (see secondary outcomes). For the evaluation of the individual domains of the risk assessment at the beginning of the intervention, the percentage of the items assessed as risky for each risk domain was determined for each participant of the intervention group. Subsequently, the mean percentages for the individual risk areas were compared by means of a one-factor variance analysis.

## Results

### Description of sample

A total of 92 informal caregivers were enrolled, of whom 47 (15 males, 32 females, mean age 72.3 y) were allocated to the intervention and 45 (16 males, 29 females, mean age 74.0 y) to the control group. Participants were recruited from the memory clinic of the University Hospital Leipzig, Department for Psychiatry and Psychotherapy (38%), from other psychiatric and neurologic hospitals and physicians (13%), outpatient care services (14%), press releases and flyer distribution (11%), practices from family doctors and occupational therapists (9%), statutory health insurances (8%), or otherwise (7%).

The baseline characteristics of the informal caregivers and relatives with dementia are presented in Table 1.

**Fig. 3:**
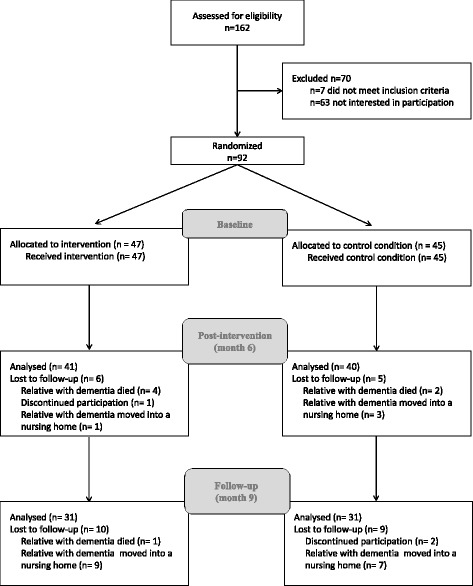
Participants flow

Informal caregivers in the intervention group showed significantly more somatization, lower psychological health-related quality of life, and stronger reaction in response to the challenging behaviors of the people with dementia than informal caregivers in the control group. There was also a trend for informal caregivers in the intervention group to be more depressed. In both groups, informal caregivers were approximately 4 years younger than people with dementia, female caregivers and male people with dementia were overrepresented, and about 50% of caregivers in the intervention (*N* = 26) and the control group (*N* = 25) received nursing support. Significantly more participants of the intervention group (*N* = 19) than of the control group (*N* = 7) visited a local caregiver support group during the intervention period. For 77% of the cases of the intervention group and for 84% cases of the control group the medical dementia diagnosis of the person suffering from dementia could be confirmed by the SISCO. For all remaining cases with an unconfirmed dementia syndrome the SISCO (SISCO) indicated at least the presence of a mild cognitive impairment. Frequency of AD, VD and bvFTD did not differ between groups. According to the SIDAM integrated Mini-Mental State Examination (MMSE_SIDAM_) [36] score, the disease severity of most of the people with dementia was in the moderate to severe stage [37]. The risk assessment of the five major risk areas of the informal caregiver of the intervention group (see above section “Description of the original REACH II program”) showed a significant effect of the factor “risk areas” (*F* = 4.49, *p* < 0.01), with the highest percentage of items at risk regarding the “perceived social support” (see Fig. 2). Simple post hoc comparison revealed that differences in the mean values between “perceived social support” and all other assessed risk areas except for “emotional and physical well-being” significantly differ from each other. The flow of participants through the study is shown in Fig. 3. All informal caregivers received the treatment they had been allocated to by the randomization procedure. At the post-intervention assessment (month 6), data were available on 41 informal caregivers in the intervention group and on 40 informal caregivers in the control group (45 and 44% of the original sample, respectively). At follow-up (month 9), 31 and 31 data sets could be analyzed (34 and 34% of the original sample, respectively).

### Feasibility and acceptance of intervention

According to the protocols of each problem-solving process and implemented contents areas, about 70% of the basic intervention modules detailed in the manual could be transferred into practice. The participants’ treatment adherence was excellent; 81% of the informal caregivers completed 10 sessions or more. Fourteen from 19 participants (see Table 3) in the intervention group, who took part in a caregiver support during intervention period followed our request to participate in a local support group.

**Table 3:** Informal carer and people with Dementia baseline characteristics

Variable	Intervention (*N* = 47) Mean ± SD or absolute (relative) frequency	Control (N = 45) Mean ± SD or absolute (relative) frequency	*P*
Informal carers			
Age (y)	72.3 ± 8.11	73.98 ± 8.15	0.331
Sex, male: female	15: 32 (32: 68)	16: 29 (36: 64)	0.826
Education (y)	13.3 ± 2.9	13.1 ± 2.6	0.693
Relation to person with dementia, spouse/partner: others	41: 6 (87: 13)	41: 4 (91: 9)	0.740
Duration of care (mo)	40.8 ± 45.1	40.8 ± 36.7	0.999
Occupational status, retiree: other	43: 4 (92: 8)	44: 1 (98: 2)	0.360
Household income [€], > 1500: < 1500	39: 6 (87: 13) ^(*N* = 45)^	31: 13 (71: 29) ^(*N* = 44)^	0.074
Burden caused by care (ZBI)	32.5 ± 12.8	28.16 ± 12.5	0.103
Somatization (PHQ 15)	8.4 ± 4.5	6.4 ± 3.9	0.022
Mental Health (PHQ 4) (total)	4.1 ± 2.8	3.2 ± 3.2	0.155
Anxiety	2.2 ± 1.6	1.8 ± 1.9	0.379
Depression	2.0 ± 1.4	1.40 ± 1.5	0.062
Perceived social support (ESSI)	18.4 ± 6.4	19.2 ± 5.7	0.509
Health-Related Quality of Life (SF-12)			
Psychological	43.7 ± 11.1	49.0 ± 10.6	0.022
Physical	43.6 ± 10.7	44.1 ± 10.7	0.844
Challenging behavior, reaction (RMBPC)	21.1 ± 9.1	16.6 ± 9.6	0.023
Nursing support, yes: no	26: 21 (55: 45)	25: 20 (46: 54)	1.000
Participation in caregiver support group, yes: no	19: 28 (45: 35)	7: 38 (18: 82)	0.008
People with dementia			
Age (y)	77.5 ± 8.3	77.2 ± 8.2	0.866
Sex, male: female	34: 13 (72: 28)	26: 19 (58: 42)	0.190
Diagnosis, AD: VD: bvFTD	38: 7: 2 (81: 15: 4)	36: 9: 0 (80: 20: 0)	0.323
General cognitive ability (MMSE_SIDAM_)	11.3 ± 9.5	13.5 ± 8.4	0.561
Dementia syndrome confirmed (SISCO), yes: no	36: 11 (77: 23)	38: 7 (84: 16)	0.687
Care level, yes: no	19: 28 (40: 60)	22: 23 (49: 51)	0.529
Challenging behavior, frequency (RMBPC), informal carer rating	22.8 ± 8.8	20.6 ± 9.0	0.234

*N* number, *P* probability, *SD* standard deviation

### Primary outcome

Burden caused by care declined very slightly in the intervention and increased very strongly in the control group from the baseline to the post-intervention assessment, as indicated by a decrease or increase in the ZBI scores (Table 4). There was a statistically significant treatment-related difference (Cohen’s d = 0.91). The presence of this effect was confirmed by the PP-analysis, and after applying the pre-specified missing value strategies (see above).

**Table 4:** Outcomes (Intention-to-treat Population)

Variable	Post-intervention (Month 6), Change from Baseline				Follow-up (Month 9), Change from Baseline			
	Intervention (N = 41) Mean ± SD	Control (*N* = 40) Mean ± SD	d_Cohen_	*P*	Intervention (N = 31) Mean ± SD	Control (*N* = 31) Mean ± SD	d_Cohen_	*P*
Primary outcome								
Burden caused by care (ZBI)	−0.425 ± 8.409	7.047 ± 8.093	0.906	0.000	2.669 ± 8.858	8.102 ± 8.576	0.623	0.017
Secondary outcome								
Somatization (PHQ 15)	−1.400 ± 3.479	1.588 ± 5.316	0,679	0.004	−0.601 ± 3.999	1.098 ± 2.773	0.502	0.057
Mental Health (PHQ 4), total	−0.659 ± 3.087	0.050 ± 3.055	0,231	0.302	−0.290 ± 2.648	0.677 ± 3.673	0.306	0.968
Anxiety	−0.342 ± 1.995	−0.025 ± 1.981	0,159	0.476	−0.194 ± 1.815	0.548 ± 2.743	0.326	0.239
Depression	−0.317 ± 1.439	0.075 ± 1.403	0,276	0.218	−0.097 ± 1.446	0.129 ± 1.432	0.157	0.214
Perceived social support (ESSI)	0.657 ± 4.860	0.214 ± 4.446	0,095	0.670	−0.550 ± 5.387	−0.742 ± 4.123	0.040	0.539
Health-Related Quality of Life (SF-12)								
Psychological	2.398 ± 8.703	−2.528 ± 8.556	0,571	0.012	3.868 ± 10.662	−4.618 ± 8.157	0.902	0.001
Physical	2.600 ± 9.960	−1.310 ± 7.711	0,443	0.052	−0.053 ± 9.591	0.191 ± 6.699	0.030	0.908
Challenging behavior, frequency (RMBPC)	−1.618 ± 7.213	1.286 ± 5.891	0,443	0.051	−1.122 ± 4.569	1.505 ± 3.991	0.614	0.022
Challenging behavior, reaction (RMBPC)	−5.461 ± 7.257	2.342 ± 6.379	1,144	0.000	−6.173 ± 7.865	3.243 ± 6.999	1,267	0.000

d_Cohen_ Cohen’s d (effect size), *N* number; *P* probability, *SD* standard deviation

### Secondary outcomes

At the post-intervention assessment (month 6), somatization (PHQ-15), reaction of the informal caregivers in response to challenging behaviors (RMBPC-24 reaction)and the frequency of challenging behaviors of the people with dementia (RMBPC-24 frequency) decreased in the intervention group and increased in the control group. Both these changes were statistically significant. Conversely, but also in the favor of the intervention group, the informal caregivers psychological health-related quality of life (SF-12) increased statistically significantly in the intervention group and declined in the control group by the time of treatment termination. However, the ESSI showed no significant changes of perceived social support for either group and also no significant differences were found between both groups with respect to global mental health (PHQ-4) and its subcomponents anxiety and depression. Finally a statistical trend was observed for a better physical health-related quality of life favoring the intervention.

At follow-up (month 9), the informal caregivers’ burden had increased in both treatment groups, but much more strongly in the control group. This difference did attain statistical significance, but compared to post-intervention assessment the effect reduced from a very great to a moderate effect (Cohen’s d = 0.62). As with the post-intervention assessment, at follow-up somatization, reaction of the informal caregivers in response to the challenging behaviors and the frequency of those behaviors of the people with dementia also declined in the intervention group and increased in the control group. The statistical trend observed in physical health-related quality of life favoring the intervention group at month 3 was no longer noted.

Regarding primary and secondary outcomes, subgroup analyses of only those cases where dementia syndromes have been confirmed by means of the SIDAM score yielded no significant different-finding pattern compared to analysis, including all enrolled cases.

## Discussion

The DE-REACH study investigated the effectiveness of an outreach, individualized and structured multicomponent intervention to support health resources of informal caregivers of people with dementia. Special attention was paid to possible effects of the intervention on the caregiver burden. DE-REACH combines several different single interventions to support informal caregivers (provision of information, psycho-education, role-playing, problem-solving, skills training to improve coping with challenging behaviors of the care recipient, cognitive strategies to reframe negative emotional responses, stress management techniques, social support and strategies to improve health behaviors). Most of the contents of the intervention were implemented. Fundamentally this indicates the feasibility of the intervention. In addition, the acceptance of the intervention was very high.

We found a very great effect on the primary outcome of the DE-REACH intervention. Caregivers of the intervention group showed a slight decrease of burden of about 0.5 scale units of the ZBI at post-intervention assessment, but the main part of the great effect on the primary outcome was the very strong increase of burden of the caregivers in the control group. Therefore a controlled study design was necessary to visualize the observed effect, which may be interpreted as a kind of stabilizing effect of the care arrangement [38]. However, the principal study objective to practically significantly relieve or reduce the burden of the informal caregivers of the intervention group was failed and the question arises as to why this was the case. Subsequent evaluation of the risk assessments of caregivers of the intervention group before the start of the intervention revealed that the risk area of the “perceived social support” was the most risky. As the telephone-based caregiver support groups of the original REACH II intervention were completely omitted, in the light of the above it seems plausible that the relief effect might have been much stronger with this intervention offer than without. This interpretation particularly seems plausible, because telephone-based support groups have shown independent effects on quality of life, feelings of burden, caregiver symptomatology, and the depression of informal caregivers [39–41]. The feasibility and effectiveness of such telephone-based support groups, which are strongly oriented to the original REACH II support groups, are also examined in an actual DE-REACH follow-up project of the first author of this study [42].

Indeed in the DE-REACH program, the “social support” intervention component was not completely omitted and the caregivers of the intervention group were definitively supported in their social networking and in their use of support offerings, and in addition, most of the caregivers of the intervention group took part in local caregiver support groups. Nevertheless, these measures may not sufficiently have met the needs for social support of the informal caregivers. The social exchange in the telephone-based support groups could have made possible a unifying “others have the same problems” experience, which could then possibly have been perceived as relief by the informal caregivers. Since the original telephone-based support groups of REACH II also had the purpose of enhancing the contents of the individual counseling sessions and the caregiver notebook by means of the social exchange between the caregivers, they would also have a reinforcing effect of other intervention components on the stress–health process of the informal caregivers.

Even if the burden of the informal caregivers is expected to increase over time (e.g. [43]), someone may claim the main part of the found stabilization effect is just a so-called ‘nocebo’ effect (from the Latin *nocere* harm, ‘*nocebo’* I will harm ‘). That is to say, in terms of a reversed placebo effect, caregivers of the control group were frustrated that they were allocated to the control condition and subsequently showed a higher level of burden. For example, Hegerl [44], who questions the great effects found for psychotherapy for mild depression, puts forward this line of argumentation. However, the nocebo effect hypothesis is contradicted by the findings that challenging behaviors of the care recipients, as a central cause of caregiver burden [3, 4], just like the (stress)reaction of the informal caregivers in response to these behaviors, increased in the control group vs. decreased in the intervention group. This effect was even greater than the effect found for the primary outcome (d = 1.14). Obviously, in the control group the weight of burden in terms of the frequency of challenging behaviors increased more than caregivers could bear, resulting in a stronger reaction in response to these behaviors (the opposite was true for the intervention group). This interpretation was confirmed by further effects on the secondary outcomes ’physical symptoms caused by stress’ (somatization) and ‘psychological health-related quality of life’ favoring the intervention group. Altogether all these findings make the nocebo-effect hypothesis very unlikely to be true or suggest that this effect, if it exists, is very small.

The fact that we could not show an effect on the depressiveness of caregivers is, in our opinion, due to the fact that we could not show a relief effect, as was the case, for example, in three implementation studies on REACH II [22–24]. All these studies revealed effects in favor of the REACH II intervention on burden, depression and the quality of life of the informal caregivers. Interestingly, all three projects had omitted the telephone-based caregiver support groups.

Furthermore, it should be stated that already after 3 months, at the follow-up assessment, the stabilization effect on the primary outcome was reduced from a great (d = 0.91) to a moderate effect size (d = 0.62). This finding was mainly due to an increase of burden in the intervention group and could be explained by an ineffective and unsustainable implementation of strategies to cope with challenging behaviors, which was a main focus of the intervention. This assumption, however, is contradicted by the finding that, compared to the assessment directly after termination of the intervention, reaction in response to challenging behaviors was even more reduced in the intervention group at follow-up assessment. This result may be interpreted that caregiver acceptance of challenging behaviors has increased. Nevertheless, the reduction of the effect size at follow-up can also be attributed to an unsustainable or insufficient social networking of the caregiver. It is possible that a large part of the found stabilizing intervention effect at post- intervention resulted from the regular contact with the DE-REACH interventionist. Plausibly this effect diminishes rapidly after the end of the intervention and the related end of the regular meetings. This interpretation would justify a continuous format of the DE-REACH intervention for implementation projects, or that after a certain number of basic sessions further consultations can be made use of as required.

### Limitations

There are some limitations to the present study that should be mentioned: First, it was restricted to a pure examination of effectiveness. Therefore as a randomized controlled trial it was conducted to a high methodical standard; however, the blinding of the independent rater could not be implemented. Second, it should be noted that the cost-effectiveness of the DE-REACH intervention was not assessed and resources utilization (e.g. use of nursing support measures) was not documented at post-intervention measurement, so it was not possible to evaluate effects of the intervention regarding this. Also the intervention change process was evaluated only basically. By change process we understand the perception of the informal caregiver and the interventionist of the importance of individual intervention components. What was especially helpful? Or what can be dispensed with?

By now we know that the intervention is fundamentally feasible and that an individualized form of counseling is well accepted by informal caregivers of people with dementia. However, a detailed knowledge about the change process or its cost-effectiveness is not available after completion of this study. Regarding future implementation studies, there are no indicators for a possible package format of the intervention, e.g. if lower doses or intensity of the intervention would be sufficient for a translation to the German health-care system. Since both mentioned aspects are significant features of phase III trials of the MRC framework [19], it is questionable whether it may be possible to proceed to phase IV of the framework (implementation) without further research efforts.

## Conclusion

Finally, and in summary, it should be noted that our results fundamentally confirm the arguments of critics of the concept of caregiver burden. For example, Purkis and Ceci [45] stated that longstanding research efforts during the past decades with the objective of relieving or reducing the burden of informal caregivers of people with dementia had been very ineffective (the DE-REACH intervention also failed to reach this goal). The authors question most research projects focusing on the isolated caregiver–recipient dyad and on the triad caregiver type, problem behavior and tailored intervention. They suggest going beyond this mainstream approach and taking care arrangements and socio–political–material relations— such as promoting the acceptance of people with dementia as part of our society (integration), political measures to promote voluntary commitment, or to simplify access to care insurance offerings— into consideration [45]. In this context the question of which system requirements stabilize care arrangements and make caregiving good [38] might be groundbreaking as a target for future health-care research.

## Abbreviations

AD: Alzheimer’s Disease
BvFTD: Behavior variant Frontotemporal Dementia
CI: Confidence Interval
d: Cohen’s d
DE-REACH: Deutsche Adaptation der Resources for Enhancing Alzheimer’s Caregiver Health II
ESSI: Enriched Social Support Instrument
MMSE: Mini-Mental State Examination
MRC: Medical Research Council
P: Probability
PHQ: Patient Health Questionnaire
PP: Per Protocol
REACH II: Resources for Enhancing Alzheimer’s Caregiver Health II
RMBPC: Revised Memory and Behavior Problem Checklist
SF-12: Short form of the SF-36 Health Survey
SIDAM: Structured Interview for Diagnosing Alzheimer-Type Dementia, Multi-stroke Dementia, and Dementias of other Etiology according to DSM-III-R and ICD-10, SIDAM
SISCO: SIDAM SCOre
SPSS: Statistical Packages for Social Science
VD: Vascular Dementia
ZBI: Zarit Burden Interview
